# Magnolol and Honokiol: Two Natural Compounds with Similar Chemical Structure but Different Physicochemical and Stability Properties

**DOI:** 10.3390/pharmaceutics13020224

**Published:** 2021-02-06

**Authors:** Iris Usach, Alessandro Alaimo, Juan Fernández, Alessandro Ambrosini, Sara Mocini, Lacramioara Ochiuz, José-Esteban Peris

**Affiliations:** 1 Department of Pharmacy and Pharmaceutical Technology and Parasitology, University of Valencia, Burjassot, 46100 Valencia, Spain; iris.usach@uv.es (I.U.); ale_ala.95@hotmail.it (A.A.); juanfernandezge@gmail.com (J.F.); alessandro.ambrosini@student.unife.it (A.A.); sara.mocini@gmail.com (S.M.); 2 Department of Pharmaceutical Technology, “Grigore T. Popa” University of Medicine and Pharmacy, 700115 Iasi, Romania; ochiuzd@yahoo.com

**Keywords:** magnolol, honokiol, solubility, stability, liposomes

## Abstract

*Magnolia* spp. extracts are known for their use in traditional Korean, Chinese, and Japanese medicine in the treatment of gastrointestinal disorders, anxiety, and allergies. Among their main components with pharmacological activity, the most relevant are magnolol and honokiol, which also show antitumoral activity. The objectives of this work were to study some physicochemical properties of both substances and their stability under different conditions of temperature, pH, and oxidation. Additionally, liposomes of honokiol (the least stable compound) were formulated and characterized. Both compounds showed pH-dependent solubility, with different solubility–pH profiles. Magnolol showed a lower solubility than honokiol at acidic pH values, but a higher solubility at alkaline pH values. The partition coefficients were similar and relatively high for both compounds (log P_o/w_ ≈ 4.5), indicating their lipophilic nature. Honokiol was less stable than magnolol, mainly at neutral and basic pH values. To improve the poor stability of honokiol, it was suitably loaded in liposomes. The obtained liposomes were small in size (175 nm), homogeneous (polydispersity index = 0.17), highly negatively charged (−11 mV), and able to incorporate high amounts of honokiol (entrapment efficiency = 93.4%). The encapsulation of honokiol in liposomes increased its stability only at alkaline pH values.

## 1. Introduction

Natural products have historically been used as remedies for the alleviation of diseases. Compared to synthetic compounds, natural bioactive compounds generally have better safety profiles, are well accepted by the patients, and are usually relatively inexpensive [1]. In this context, extracts from the bark of *Magnolia* species, such as *M. officinalis* and *M. obovata*, are widely used in traditional Korean, Chinese, and Japanese herbal medicine for the treatment of gastrointestinal disorders, anxiety, and allergies [2]. Other reported actions include anti-inflammatory [3,4,5], antimicrobial [6,7,8], anti-oxidative [9,10,11], neuroprotective [12], anti-thrombotic [13], and anti-depressant [14,15] properties. 

**Figure 1:**
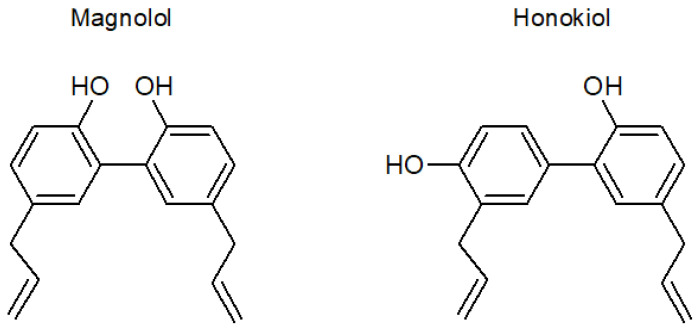
Structural formulas of magnolol and honokiol.

The main substances responsible for the beneficial features of the *Magnolia* bark extract [16] are the neolignans magnolol and honokiol. Magnolol (5, 5′-diallyl-2, 2′-dihydroxybiphenyl) and honokiol (3,5′-diallyl-4,2′-dihydroxybiphenyl) are positional isomers (Figure 1) with biphenolic groups [17,18]. The highest content of both substances is found in the roots of trees, and its concentration in the extracts varies widely depending on various environmental factors such as the area of origin, altitude of the cultivar, and age of the tree [19]. 

In addition to the pharmacological actions described above, several preclinical studies have shown that magnolol and honokiol are also effective against different types of cancer such as lung, prostate, breast, gall bladder, colon, skin, and hepatocellular carcinoma [20,21]. However, their potential use in clinical applications is restricted by their very low oral bioavailability [17,22,23]

Magnolol and honokiol are commercially available as pure substances isolated from magnolia extracts. In the solid state, both compounds show a high degree of crystallinity, with melting points around 102 °C (magnolol) and 87 °C (honokiol) [24,25,26]. Although both substances show similar pharmacological activities, knowing the differences between their physicochemical and stability properties is of interest for the development of medicines containing these active substances. For example, the low bioavailability of both compounds has been attributed to their very low water solubility. In this regard, a solubility–pH profile might suggest which are the more suitable pH values for solubilization. 

The use of some natural compounds can be limited due to several factors, including chemical instability. In the case of magnolol and honokiol, their stability can be affected by hydrolytic and oxidizing conditions due to their biphenolic structure. Their incorporation in nanocarriers represents a suitable approach to overcome stability limitations [27]. Liposomes are considered, due to their biological and technological features, highly versatile drug-nanocarrier systems [28]. They offer many advantages such as increased apparent solubility and improved chemical stability of natural compounds [27]. Liposomes increase drug half-life, improve the therapeutic index, and are considered biocompatible and biodegradable [29,30]. It has been also described that liposomes can enhance the activity of natural compounds, such as citral and *thymus* essential oils, which improve their antifungal and antioxidant activity, respectively, when formulated in liposomes [31,32].

The objective of this work was to study some physicochemical properties of magnolol and honokiol, such as water solubility and octanol–water distribution at different pH values, as well as to evaluate their stability under different conditions of temperature, pH, and oxidation. As honokiol showed poorer stability than magnolol, it was loaded in liposomes, characterized according to their morphology, size distribution, zeta potential, and entrapment efficiency. The stability of honokiol-encapsulated liposomes was evaluated and compared with the unencapsulated compound.

## 2. Materials and Methods

### 2.1. Reagents

Magnolol and honokiol (98% purity) were acquired from New Natural Biotechnology (Shanghai, China). Phosphotungstic acid solution, sodium phosphate monobasic and potassium phosphate monobasic were purchased from Sigma-Aldrich (Madrid, Spain). *N*-octanol, acetonitrile, ethyl alcohol, and potassium chloride were obtained from VWR chemicals (Barcelona, Spain). Hydrochloric acid 37% and sodium hydroxide were purchased from Scharlab (Barcelona, Spain). Hydrogen peroxide 30% and boric acid were obtained from Merck (Barcelona, Spain). Phospholipon 90 G was provided by Lipoid GmbH (Ludwigshafen, Germany).

### 2.2. Analytical Method

Magnolol and honokiol were quantified by high performance liquid chromatography assay (HPLC) with ultraviolet (UV) detection at 290 nm. The HPLC equipment consisted of an Agilent chromatograph, a G1379A degasser, a G1310A pump, a G1329A automatic injector, and a G1314A variable wavelength spectrophotometric detector. The column was a Waters “Nova-Pack” C_18_ (4 µm, 3.9 mm × 150 mm), and the mobile phase consisted of a mixture of acetonitrile and 25 mM sodium phosphate monobasic buffer, pH 4.6 (60/40, *v*/*v*). The injection volume was 25 µL, and the flow rate was 1 mL/min.

### 2.3. Method Validation 

The calibration curves (peak area versus nominal concentration) were constructed using a least square linear regression analysis for the calculation of the slope, intercept, and correlation coefficient. The accuracy (bias) and precision (relative standard deviation; RSD) of the assay were determined from magnolol and honokiol standards prepared at four concentrations (1, 10, 50, and 75 µg/mL).

The limit of quantification (LOD) was estimated as the concentration of magnolol or honokiol giving rise to a peak whose height is 10 times the signal-to-noise ratio. The lower limit of quantification (LLOQ) was determined as the concentration of the lower standard with accuracy within 80–120% and RSD within 20%.

### 2.4. Determination of Solubility in Water 

The aqueous solubility was evaluated using the shake-flask method. Briefly, approximately 40 mg of magnolol or honokiol were added to a volume of 10 mL of distilled water. The mixture was kept in constant agitation for a total period of 24–48 h. Samples were taken at 24 and 48 h, centrifuged for 2 min at 10× *g*, and the concentration of the dissolved compound was determined in the supernatant using the analytical method described above. The 48-h sample was used to confirm that a dissolution equilibrium had been reached after 24 h of agitation.

Solubility at different pH values (1.2, 4.5, 6.8, 7.4, 8, 9, and 10) was determined at 37 °C after 24 h of agitation. The composition of the solutions is described in USP 35 [33] and the European Pharmacopeia 7.0 [34]. Briefly, the pH 1.2 solution was prepared with diluted hydrochloric acid, the solutions with pH values from 4.5 to 7.4 were phosphate buffers, and the solutions with pH 9 and 10 were borate buffers. Additionally, two pH 8 solutions were prepared using phosphate and borate buffers.

### 2.5. Determination of Octanol-Water Distribution Coefficients

Octanol–water distribution coefficients (D_o/w_) of magnolol and honokiol were determined by dissolving each compound in n-octanol, previously saturated with buffer (pH 1.2, 4.5, 6.8, and 7.4) or water, using glass vials. Buffers and water saturated with n-octanol were added to the corresponding vials, which were stirred for approximately 12 h at room temperature. Thereafter, the organic and the aqueous phases were separated by centrifuging the contents of the vials (2 min at 8000× *g*), and the concentration of each compound in both phases was determined by HPLC. The distribution coefficient was obtained as the quotient of the concentrations in the n-octanol and aqueous phases.

### 2.6. Forced-Degradation (Stress Testing) Studies

The stability of both natural products was evaluated in forced conditions at 60 °C using different pH values. Magnolol and honokiol solutions were prepared in 0.1 M HCl, 0.1 M NaOH, and buffers (pH 7.4, 8, 9, and 10). These solutions were kept at 60 °C for 24 h and the concentration of remaining magnolol and honokiol was determined.

The stability was also evaluated at room temperature and 37 °C (pH 1.2, 4.5, 6.8, 7.4, 8, 9, and 10) after an incubation period of 30 days.

Additionally, stability was examined under oxidizing conditions using 3% H_2_O_2_. These samples were kept at room temperature, 37 °C, and 60 °C, for 24 h. The concentration of samples was expressed as a percentage of the initial concentration.

### 2.7. Liposome Preparation

Honokiol-loaded liposomes were formulated with Phospholipon 90 G (10 mg/mL), honokiol (2 mg/mL), ethanol (0.5 mL), and bidistilled water (5 mL). The dispersion was warmed at 50 °C and sonicated (2 cycles, 5 s on and 2 s off, 60% amplitude) with an ultrasonic disintegrator (CY-500, Optic Ivymen system, Barcelona, Spain) to homogenize the preparation. To avoid a high increase in the temperature of the mixture as a consequence of the sonication process, the vial containing the mixture was placed in a container with water at room temperature. The temperature of the mixture at the end of the sonication was around 52 °C. Empty liposomes (without honokiol) were also prepared and used as reference in the characterization of honokiol-loaded liposomes.

### 2.8. Liposome Characterization

Transmission electron microscopy (TEM) confirmed the formation of liposomes and their morphology. The samples were stained with 2% phosphotungstic acid aqueous solution and examined under a JEM-1010 (Jeol Europe, Paris, France) transmission electron microscope equipped with an AMT RX80 digital camera and the AmtV602 software, version 602.579, at an accelerating voltage of 80 kV.

Mean diameter (MD) and polydispersity index (PI) were determined by photon correlation spectroscopy using a Zetasizer nano (Malvern Instruments, Worcestershire, UK). The same equipment was also used to measure the zeta potential (ZP) by means of the M3-PALS (phase analysis light scattering) technique, which measures the particle electrophoretic mobility.

The total concentration of honokiol in the liposome suspension was determined by HPLC, and drug recovery (DR %) was calculated according to the following equation:

(1) $$\mathrm{DR}\;(\%)\;=\;\frac{\mathrm{Total}\;\mathrm{conc}.\;\mathrm{in}\;\mathrm{liposome}\;\mathrm{suspension}}{\mathrm{Theoretical}\;\mathrm{conc}.}\;\times \;100$$

where the theoretical concentration is 2 mg/mL.

To evaluate the percentage of honokiol actually encapsulated in the liposome suspension, an aliquot was dialyzed against water. The time required to reach the equilibrium in the diffusion of drug molecules from the inside and the outside of tube dialysis was determined before, with the following experiment: 1 mL of a solution of honokiol in water (40 µg/mL) was introduced into the dialysis membrane (Spectra/Por^®^ membranes: 12–14 kDa MW cut-off, 3 nm pore size; Spectrum Laboratories Inc., DG Breda, The Netherlands), which was immersed in 100 mL of water under continuous stirring for 48 h at room temperature. At specific time intervals, 0.5 mL of the external water was taken, mixed with 0.5 mL of acetonitrile, and injected into the chromatograph to measure the drug concentration outside the dialysis tube. The dialysis equilibrium was assumed to be reached when the concentration remained constant for subsequent samples. Additionally, it was confirmed that the equilibrium had been reached by sampling the inside of the dialysis tube and determining the concentration of the drug at the end of the assay.

To determine the concentration of the free (unencapsulated) drug, 1 mL of liposomes containing honokiol was introduced into the dialysis bag, which was immersed in 400 mL of water and subjected to agitation. After reaching dialysis equilibrium, a sample of 0.5 mL of the exterior aqueous medium was taken and added to 0.5 mL of acetonitrile. The mixture was injected into the chromatograph to determine the concentration of honokiol in the external aqueous medium, which is assumed to be equal to the free (unencapsulated) concentration of honokiol in the liposome suspension. Additionally, the total concentration of honokiol inside the dialysis tube was determined by injecting a sample of the liposome suspension, mixed with the same volume of acetonitrile, into the chromatograph. The encapsulated drug (ED %) was calculated with the following equation:

(2) $$\mathrm{ED}\;(\%)\;=\;\frac{\mathrm{Conc}.\;\mathrm{inside}\;\mathrm{the}\;\mathrm{liposomes}}{\mathrm{Total}\;\mathrm{conc}.}\;=\;\frac{\mathrm{Total}\;\mathrm{conc}.\;\mathrm{inside}\;\mathrm{dyalisis}\;\mathrm{tube}\;-\mathrm{Free}\;\mathrm{conc}.}{\mathrm{Total}\;\mathrm{conc}.\;\mathrm{inside}\;\mathrm{dyalisis}\;\mathrm{tube}}\;\times \;100$$

Entrapment efficiency (EE %) was obtained as follows:

(3) $$\mathrm{EE}\;(\%)\;=\;\frac{\mathrm{Conc}.\;\mathrm{inside}\;\mathrm{the}\;\mathrm{liposomes}}{\mathrm{Theoretical}\;\mathrm{conc}.}\;=\;\frac{\mathrm{DR}\;\left(\%\right)\;\times \;\mathrm{ED}\;\left(\%\right)}{100}$$

### 2.9. Stability Studies of Honokiol-Loaded Liposomes 

In order to compare the stability of honokiol formulated in liposomes with the stability of the raw compound, liposomes were kept at room temperature and 37 °C for 30 days using the buffers previously described (pH 1.2, 4.5, 6.8, 7.4, 8, 9, and 10). The residual concentration of honokiol was evaluated by HPLC and expressed as the percentage of the initial concentration.

The stability of honokiol formulated in liposomes was studied using diluted and undiluted liposomes. Liposomes were diluted to avoid phenomena such as aggregation and precipitation.

### 2.10. Statistical Analysis

Data are presented as mean ± standard deviation (SD). The Student’s *t*-test was used for comparisons of two groups, and *p* values of <0.05 were considered statistically significant. All calculations were performed with IBM SPSS Statistics 24 (SPSS Inc., Chicago, IL, USA).

## 3. Results

### 3.1. Assay Validation

**Figure 2:**
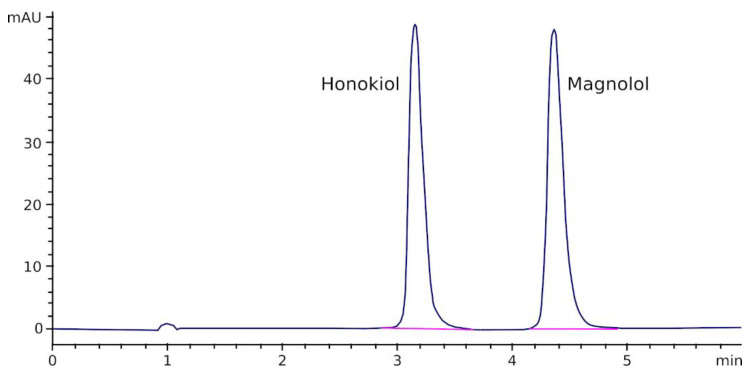
Chromatogram obtained after the injection of a mixture containing magnolol and honokiol (10 µg/mL each) into the HPLC equipment.

Figure 2 shows a chromatogram obtained after injecting a solution of magnolol and honokiol in acetonitrile/water (50/50, *v*/*v*) into the chromatograph. As can be appreciated, both peaks are completely separated and no interfering peaks were observed at, or near, the retention time of magnolol and honokiol.

A linear relationship was found between the magnolol and honokiol peak area and their concentrations in standards in the range of 0.1–100 µg/mL (magnolol: Peak area = 41.74 × Conc(µg/mL)—12.31; honokiol: Peak area = 69.97 × Conc(µg/mL)—7.43; r > 0.9992 for both relationships). LOD was approximately 0.01 µg/mL, and LLOQ was established at 0.1 µg/mL. Bias and RSD values of the method were lower than 10% and 3%, respectively, for both compounds (Table 1).

### 3.2. Solubility and Partition Coefficient

A preliminary solubility test of both compounds in water was performed by keeping the mixtures under agitation for 24 and 48 h. The results obtained at both sampling times were similar, indicating that a mixing period of 24 h was sufficient for reaching the solubility equilibrium. For this reason, a mixing time of 24 h was used to determine the solubility of magnolol and honokiol in different buffers.

The solubility of magnolol in water at room temperature obtained in the preliminary solubility test was lower than the solubility of honokiol in the same conditions: 12.5 ± 0.6 µg/mL vs. 50.6 ± 1.2 µg/mL. 

**Figure 3:**
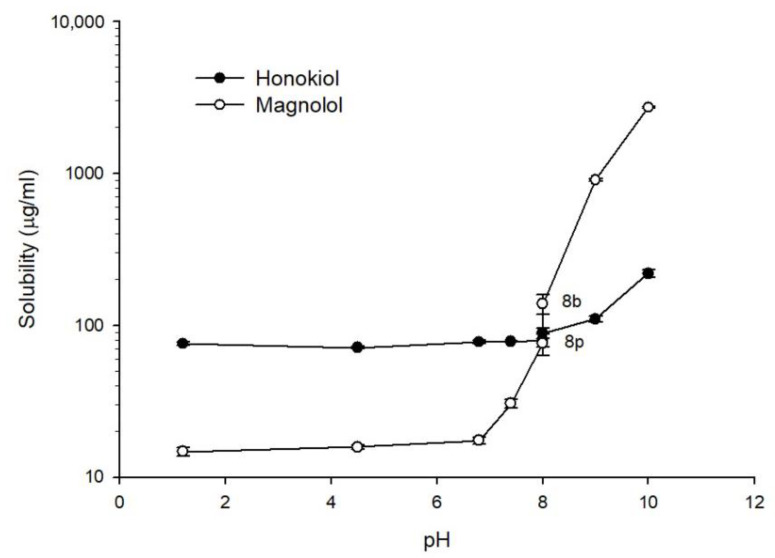
Solubility of magnolol (○) and honokiol (●) as a function of pH at 37 °C. pH 8p: phosphate buffer; pH 8b: borate buffer.

Figure 3 shows the solubility of magnolol and honokiol as a function of pH at 37 °C. For pH values lower than 7.4, the solubility of honokiol (approx. 75 µg/mL) was higher than the solubility of magnolol (approx. 16 µg/mL). An increase of the solubility of magnolol as a function of pH was observed starting at pH 7.4. The solubility of magnolol at pH 8 was dependent on the composition of the buffer, being higher for borate buffer (138 µg/mL) than phosphate buffer (76 µg/mL). At pH 9 and 10, the solubility of magnolol was higher than the solubility of honokiol. The pH-dependent solubility of both compounds can be related to the ionization at alkaline pH values given their phenolic structures.

The log D_o/w_ values obtained in the pH range from 1.2 to 7.4 were similar for both compounds (Table 2). When the aqueous phase was water instead of buffer, a slightly lower value was obtained for magnolol.

### 3.3. Stability of Magnolol and Honokiol 

**Figure 4:**
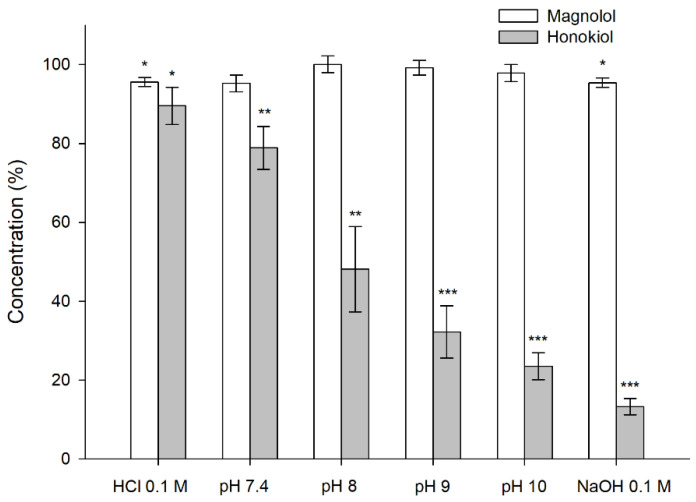
Stability of magnolol and honokiol in 0.1 N HCl, 0.1 N NaOH, and different pH buffers after 24 h at 60 °C. Data are expressed as the percentage of the initial concentration. * *p* < 0.05, ** *p* < 0.01, *** *p* < 0.001.

**Figure 5:**
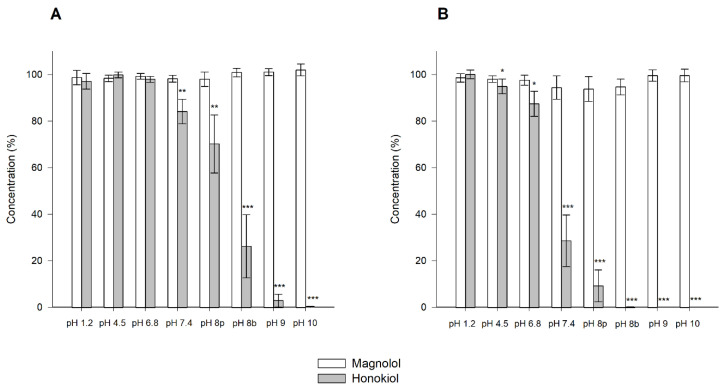
Stability of magnolol and honokiol as a function of pH after 30 days at room temperature (**A**) and 37 °C (**B**). Data are expressed as the percentage of the initial concentration. pH 8p: phosphate buffer; pH 8b: borate buffer. * *p* < 0.05, ** *p* < 0.01, *** *p* < 0.001.

The results obtained in the stability tests carried out at 60 °C for 24 h, with different pH values, showed that magnolol was relatively stable under these conditions, whereas honokiol showed a pH-dependent degradation (Figure 4). When the studies were performed at room temperature and 37 °C for one month, the degradation of honokiol was also evident at alkaline pH values (Figure 5). At pH 7.4, the concentration of honokiol was 84% of the initial concentration at room temperature, and 29% at 37 °C. In the case of pH values lower than 7.4, no degradation of honokiol was observed at room temperature. However, a decrease in the initial concentration was detected at 37 °C (pH 4.5 and 6.8). The values obtained with phosphate and borate buffers at pH 8 indicate that borate buffer increases the degradation of honokiol.

**Figure 6:**
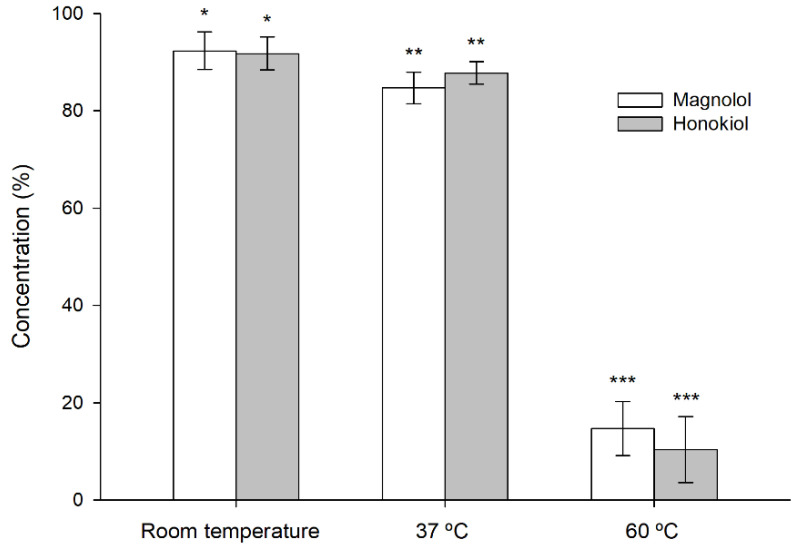
Stability of magnolol and honokiol after 24 h at room temperature, 37 °C, and 60 °C, under oxidizing conditions (3% hydrogen peroxide). Data are expressed as the percentage of the initial concentration. * *p* < 0.05, ** *p* < 0.01, *** *p* < 0.001.

The stability of magnolol and honokiol was affected by an oxidizing environment consisting of 3% hydrogen peroxide (Figure 6). The degradation increased with the temperature and was similar for both compounds, unlike what happened in the studies with different pH buffers.

### 3.4. Liposome Preparation and Characterization

**Figure 7:**
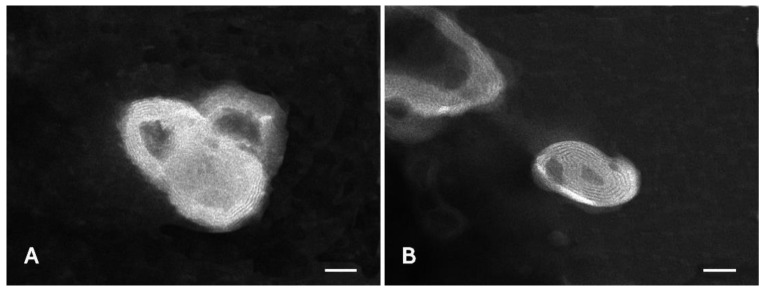
Transmission electron microscopy (TEM) images of empty liposomes (**A**) and honokiol-loaded liposomes (**B**). Bars correspond to 50 nm.

Liposomes loaded with honokiol were mainly multilamellar, as detected by TEM analyses (Figure 7). The liposomes were small in size, spherical in shape, and slightly aggregated. Empty liposomes were also prepared in order to assess the effect of honokiol on liposome assembly.

The physicochemical properties of liposomes were evaluated measuring the mean diameter (MD), polydispersity index (PI), and zeta potential (ZP) (Table 3). The empty liposomes were slightly bigger (222 nm) than those loaded with honokiol (175 nm), this difference being statistically significant. The incorporation of honokiol in liposomes led to a significant increase in the homogeneity of the systems, as the PI decreased from 0.38 to 0.17 (*p* < 0.01). The zeta potential was negative for all the liposomes, which is predictive of good stability when stored, due to repulsive forces between particles able to avoid their aggregation and fusion. The analysis by HPLC of the honokiol content in liposome suspension gave rise to a DR value of 93.4%.

**Figure 8:**
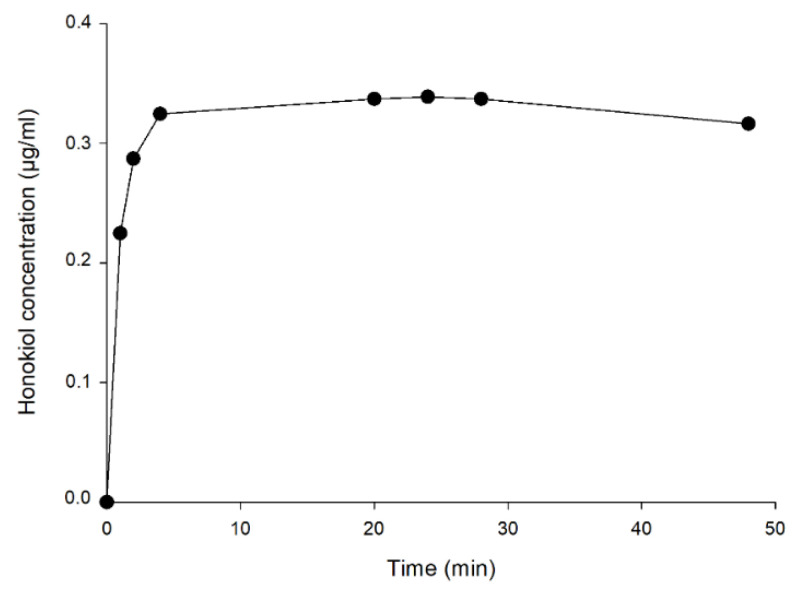
Concentrations of honokiol in the external aqueous medium during the dialysis test.

Figure 8 shows the concentrations of honokiol obtained in the external aqueous medium during the dialysis assay to determine the time to reach the equilibrium between the internal and external concentrations. The external concentration remained stable from 4 h until the end of the experiment (48 h), being approximately 0.32 µg/mL. The concentration inside the dialysis tube at the end of the experiment (0.30 µg/mL) was close to the concentration in the external medium (0.32 µg/mL), which indicates that an equilibrium between both concentrations had been reached. From the results of these assays, it was estimated that 4 h are required for reaching the equilibrium.

In the dialysis test with liposomes, the concentration of honokiol in the water outside the dialysis tube at 4 h, determined by HPLC, was assumed to be equal to the concentration of free (unencapsulated) compound in the inner water (which contained the liposomes). The total concentration of honokiol inside the dialysis tube was also determined at the end of the experiment to obtain the ED, which was 99.9% (Equation (2)). Finally, an entrapment efficiency (EE) of 93.4% was calculated using Equation (3).

### 3.5. Stability of Honokiol-Loaded Liposomes

**Figure 9:**
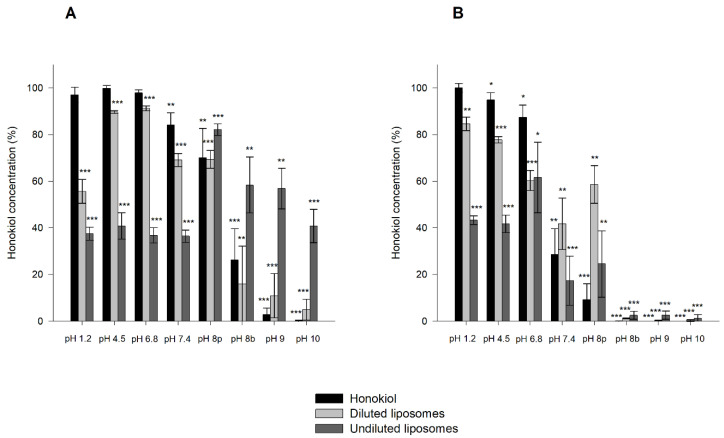
Stability of honokiol and honokiol-loaded liposomes (diluted and undiluted) after 30 days of incubation at room temperature (**A**) and 37 °C (**B**). Data are expressed as the percentage of the initial concentration. pH 8p: phosphate buffer; pH 8b: borate buffer. * *p* < 0.05, ** *p* < 0.01, *** *p* < 0.001.

The results obtained in the stability tests of honokiol-loaded liposomes at room temperature and 37 °C in solutions of different pH values are shown in Figure 9. For pH values in the range of 1.2 to 7.4, honokiol encapsulated in liposomes was less stable than unencapsulated liposomes at room temperature, and the same occurred in the pH range of 1.2 to 6.8 at 37 °C. Additionally, the undiluted liposomes were less stable than diluted ones. At a pH of 8 or higher, undiluted liposomes were more stable than unencapsulated honokiol at room temperature. In the case of the studies performed at 37 °C, these comparisons are difficult to make because honokiol was almost totally degraded in all assayed formulations. However, at 37 °C and pH 8 (phosphate buffer), the diluted and undiluted liposomes were more stable than unencapsulated honokiol. The degradation of honokiol and liposome-encapsulated honokiol at room temperature and 37 °C was larger at pH 8 when the buffer contained borate instead of phosphate. In summary, the encapsulation of honokiol in liposomes increases its stability at alkaline pH values.

## 4. Discussion

Among the components of *Magnolia officinalis* extracts with pharmacological activity, the most relevant are magnolol and honokiol. These natural compounds have attracted great interest in recent years for their potential therapeutic applications. However, a limitation in the use of magnolol and honokiol is their poor water solubility, which can greatly restrict gastrointestinal absorption and bioavailability. The water solubilities of magnolol and honokiol at room temperature obtained in this work were 12.5 and 50.6 µg/mL, respectively. However, the solubility of both compounds can be affected by the medium pH, since their molecules contain phenolic groups that can be ionized at alkaline pH values. In fact, our results showed a remarkable increase of the magnolol solubility starting at pH 7.4, reaching a solubility of approximately 2700 µg/mL at pH 10. The increase in the solubility of honokiol with the pH started at pH 9 and was less pronounced, with a solubility of approximately 220 µg/mL at pH 10. These studies also showed that borate buffer increased the solubility in comparison with phosphate buffer (pH 8), mainly in the case of magnolol. The difference in the solubility profile as a function of the pH for both compounds can be explained considering their pKa values (magnolol pKa values: 7.10 and 10.58; honokiol pKa values: 9.64 and 10.71) [35]. Using the obtained solubility–pH data, the pKa of magnolol can be estimated from the intersection of the straight lines corresponding to pH 1.2–6.8 and to pH 7.4–10 in Figure 3 [36]. This graphical estimation gives a pKa value of 7.2 for magnolol, which is close to the reported value of 7.1. In the case of honokiol, the same procedure gives an estimated pKa of 8.5, which is smaller than the reported value of 9.64. This discrepancy is probably due to the reduced number of values for the second straight line (pH 9 and 10). A previous study of magnolol solubility [37], performed using three different pH values (1.2, 3.5, and 7.4) and 2 h of agitation, reported solubility values (3.25, 4.95, and 0.04 µg/mL, respectively) lower than those obtained in this study (14.75 µg/mL for pH 1.2 and 30.51 µg/mL for pH 7.4). The different solubility values obtained in both studies could be related to the different agitation time, which was shorter in the study of Lee et al. (2 h vs. 24 h). Another discrepancy between both studies is that the solubility at pH 7.4 was, in comparison to the solubility at pH 1.2, approximately 99-fold lower and 2-fold higher in the study by Lee et al. and the present one, respectively. In the case of honokiol, no data about its solubility at different pH values have been found in the available literature. A solubility of 14 µg/mL of honokiol in water has been reported [38], which is lower than that obtained in this work (50.6 µg/mL). Again, the discrepancy in these values could be related to the agitation time (4 h vs. 24 h).

The log D_o/w_ values were obtained in the pH range from 1.2 to 7.4, which covers the pH of the gastro-intestinal tract. The obtained values were very similar for both compounds, with no differences depending on the pH, although a slight decrease was observed for pH 7.4. The log D_o/w_ values obtained at the acidic pHs can be interpreted as the logarithm of the partition coefficient (log P_o/w_), since it can be assumed that only unionized species are present in the organic and aqueous phase, given the pKa values of magnolol and honokiol. Consequently, a log P_o/w_ of approximately 4.5 has been obtained for these compounds, indicating their high hydrophobicity. The obtained log P_o/w_ value of 4.5 is slightly higher than the theoretical estimations available in the literature (3.94 and 4.20 for magnolol and honokiol, respectively), obtained through the use of a computer program [39]. The high lipophilicity of these substances suggests a rapid absorption by passive diffusion, but their low solubility limits the amount of dissolved molecules available for the absorption. A review of the literature showed that a log P_o/w_ between 1 and 3 is optimal for in vivo pharmacokinetics, although there are successful drugs that do not fall within this lipophilic range [40]. Furthermore, Lipinski’s rule of five predicts poor absorption and permeation for compounds with a log P_o/w_ greater than 5 [41], which is close to the value obtained for magnolol and honokiol. All this suggests that some technological strategy, such as formulation with surfactants or nanocarriers, will be necessary to achieve therapeutic levels of magnolol and honokiol in blood and tissues when administered extravascularly.

The stability studies at different pH and temperature values showed that honokiol was less stable than magnolol. This was an unexpected result considering the similar structure of both compounds (Figure 1) and could address the selection of magnolol against honokiol in the case of aqueous formulations with non-acidic pH. No differences between both compounds were observed with regard to their stability under oxidizing conditions, which indicates a similar antioxidant activity. Although the pH of the oxidizing solution was 3.5, the instability of magnolol and honokiol was higher than that observed in similar conditions of pH and temperature without the oxidizing agent, which indicates that the oxidizing environment rather than the acidic pH was the main factor responsible for the instability of these compounds in the experiments with hydrogen peroxide.

Honokiol-loaded liposomes were prepared by direct sonication, which avoids the use of organic solvents. Honokiol was encapsulated in liposomes with the aim of improving its stability in aqueous media. The obtained liposomes were fairly spherical, small in size, with several layers, and able to incorporate a very high proportion of the amount of honokiol initially used for the preparation (EE = 93.39%).

The liposomes containing honokiol were smaller than empty liposomes, with a lower PI and a larger negative ZP. All these properties indicate that honokiol improves the homogeneity of the liposomes and the stability of the colloidal system, preventing the aggregation of the dispersed liposomes. The formulation of honokiol in liposomes enables a remarkable increase in its solubility. In fact, the concentration of the active substance in the liposomal preparation was 2 mg/mL. The stability studies showed that honokiol formulated in liposomes was less stable than aqueous solutions of the raw material at acidic pH values, which suggests some chemical interaction between honokiol and the liposome phospholipids. In fact, the stability was higher in the case of diluted liposomes, with a lower concentration of phospholipids. However, liposomes increased the stability of honokiol at basic pH values, this effect being more evident in the case of undiluted liposomes.

## 5. Conclusions

The results of this study showed that magnolol and honokiol are highly hydrophobic compounds with poor water solubility that limits their clinical applications. Both compounds exhibited pH-dependent solubility, with different solubility–pH profiles. Magnolol showed a lower solubility than honokiol at acidic pH values, but a higher solubility at alkaline pH values. Honokiol was less stable than magnolol, mainly at neutral and basic pH values. The incorporation of honokiol in liposomes improved its stability at basic pH values and may be considered as a promising formulation for the therapeutic administration of this compound.

## Figures and Tables

**Table 1:** Precision and accuracy for the determination of magnolol and honokiol (*n* = 4).

Nominal Concentration (µg/mL)	Magnolol			Honokiol		
	Observed Concentration (µg/mL)	RSD (%)	Bias (%)	Observed Concentration (µg/mL)	RSD (%)	Bias (%)
1	1.08	1.0	7.9	1.09	1.9	8.6
10	10.70	1.3	7.0	10.41	1.2	4.1
50	49.32	3.0	−1.4	49.78	1.8	−0.4
75	76.37	0.4	1.8	78.15	0.5	4.2

**Table 2:** Log D_o/w_ values (mean ± SD, *n* = 4) of magnolol and honokiol determined using n-octanol (organic phase) and different pH buffers or water (aqueous phase).

Aqueous Phase	Log D_o/w_	
	Magnolol	Honokiol
pH 1.2	4.50 ± 0.10	4.48 ± 0.05
pH 4.5	4.55 ± 0.11	4.50 ± 0.08
pH 6.8	4.48 ± 0.06	4.48 ± 0.04
pH 7.4	4.30 ± 0.08	4.28 ± 0.05
H_2_O	4.07 ± 0.09	4.27 ± 0.06

**Table 3:** The mean diameter (MD), polydispersity index (PI), and zeta potential (ZP) of empty and honokiol-loaded liposomes. The mean values ± standard deviations are reported (*n* = 3).

Sample	MD (nm)	PI	ZP (mV)
Empty liposomes	222 ± 7	0.38	−7.0 ± 0.4
Honokiol-loaded liposomes	175 ± 3	0.17	−11.0 ± 1.4

## Data Availability

Not applicable.
